# Supplementary material for: Light-triggered release of conventional local anesthetics from a macromolecular prodrug for on-demand local anesthesia
Source: Nat Commun. 2020 May 8;11:2323. doi: 10.1038/s41467-020-16177-w (PMC7210304; doi:10.1038/s41467-020-16177-w)
Supplement: Supplementary file 1 — Supplementary Information [file 41467_2020_16177_MOESM1_ESM.pdf]

Supplementary Information

Light-Triggered Release of Conventional Local Anesthetics from a Macromolecular Prodrug for on-Demand Local Anesthesia

Wei Zhang,[1] Tianjiao Ji,[1] Yang Li,[1] Yueqin Zheng,[1] Manisha Mehta,[1] Chao Zhao,[1] Andong Liu[1] and Daniel S. Kohane*[1]

[1]Laboratory for Biomaterials and Drug Delivery, The Department of Anesthesiology, Critical Care and Pain Medicine, Boston Children's Hospital, Harvard Medical School, Boston, MA, USA

*E-mail: daniel.kohane@childrens.harvard.edu

Supplementary Methods

Materials and Reagents. The chemical and solvents were used as received: 7-diethylamino-4-methylcoumarin (Sigma-Aldrich, 99%), selenium dioxide (Sigma-Aldrich, 99.8%), celite (Sigma-Aldrich), nitromethane (Alfa Aesar, 98%), *N,N,N'N'*-tetramethylethylenediamine (Alfa Aesar, 99%), acetic acid (Sigma-Aldrich, 99%), zinc (purum, powder, Sigma-Aldrich), sodium carbonate (Sigma-Aldrich, 99%), sodium chloride (Sigma-Aldrich, 99%), sodium sulfate anhydrous (Sigma-Aldrich, 99%), 4-azidobenzoic acid (TCI America, 97%), triethylamine (TCI America, 99%), *O*-(7-Azabenzotriazol-1-yl)-*N,N,N',N'*-tetramethyluronium hexafluorophosphate (HATU, Chem-Impex International, 99%), triphosgene (Chem-Impex International, 99%), *N,N*-diisopropylethylamine (DIPEA, Sigma-Aldrich, 99%), 1,8-diazabicyclo[5.4.0]undec-7-ene (Alfa Aesar, 99%), Poloxamer 407 (from BASF), sodium hydride (Sigma-Aldrich, dry, 90%), propargyl bromide (Sigma-Aldrich, 80wt% in toluene), Copper (II) sulfate pentahydrate (Sigma-Aldrich, 99%), sodium (+)-L-ascorbate (Sigma-Aldrich, 99%), sodium borohydride (Sigma-Aldrich, 98%), tetracaine (Sigma-Aldrich, 98%), tetracaine hydrochloride (Sigma-Aldrich, meets United States Pharmacopeia specifications), dichloromethane (Sigma-Aldrich, 99% or anhydrous), hexane (Sigma-Aldrich, mixture of isomers, 99%), ethyl acetate (Sigma-Aldrich, 99%), dioxane (Sigma-Aldrich, 99%), tetrahydrofuran (Sigma-Aldrich, 99%), diethyl ether (Sigma-Aldrich, 99%), methanol (Sigma-Aldrich, 99%), *d*-chloroform (Cambridge Isotope Laboratories Inc., 99%). NO_2-CM-OH was synthesized according previously reported method with some modifications.[1]

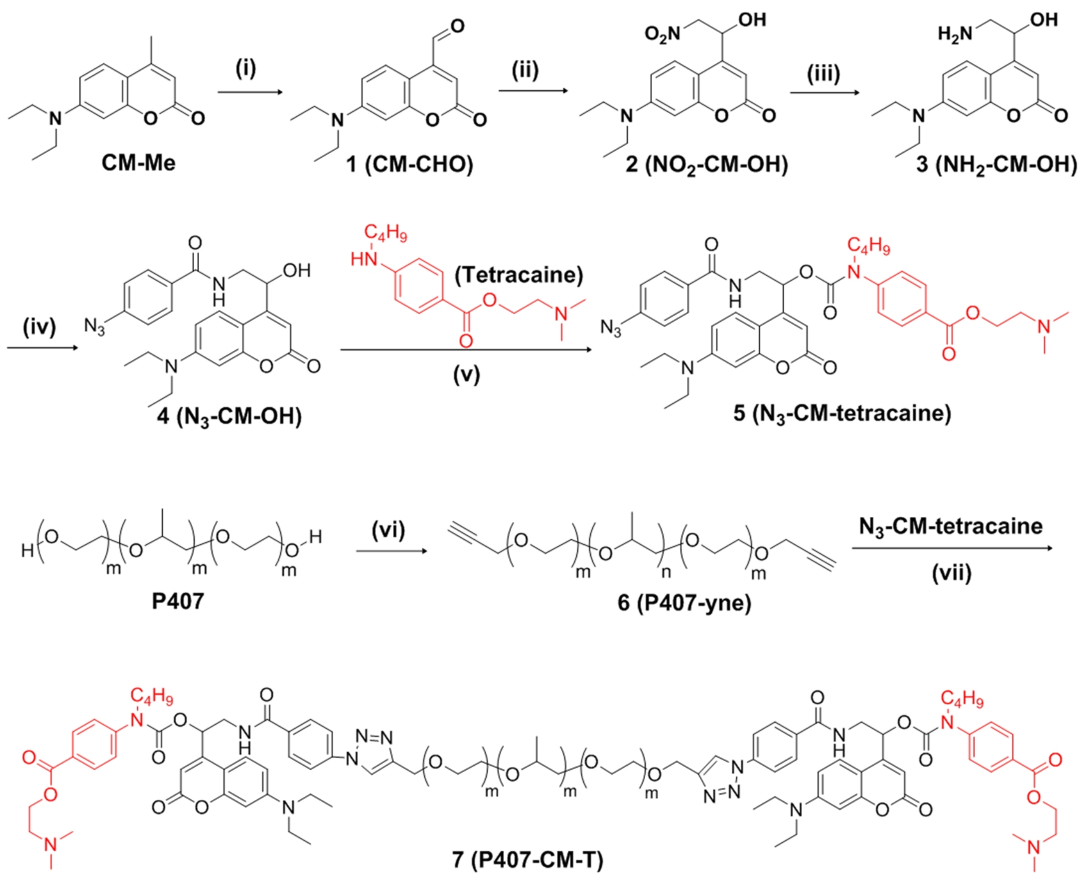

(i) SeO_2, 1,4-dioxane, reflux; (ii) CH_3NO_2, *N,N,N'N'*-tetramethylethylenediamine, THF; (iii) Zn, acetic acid; (iv) 4-azidobenzoic acid, DIC, DMAP, DCM; (v) tetracaine, triphosgene, DIPEA, DBU, DCM; (vi) sodium hydride , THF; (vii) Copper (II) sulfate pentahydrate, (+)-sodium L-ascorbate, methanol/H_2O.

Supplementary Figure 1. Synthetic route to P407-CM-T.

(i) Synthesis of Compound 1 (CM-CHO). 7-Diethylamino-4-methylcoumarin (2.31 g, 10 mmol) was dissolved in 1,4-dioxane (50 mL). Selenium dioxide (2.00 g, 18 mmol) was added. The mixture was refluxed overnight before filtered through celite. The filtrate was concentrated and purified by silica gel chromatography (dichloromethane: hexane 3:1) to give the product as a red crystal (860 mg, 35%). ^{1}H NMR ($CDCl_3$, 400MHz, ppm, δ) 9.95 (s, 1H), 8.21-8.19 (d, 1H), 6.60-6.57 (d, 1H), 6.46 (s, 1H), 6.37 (s, 1H), 3.40-3.34 (m, 4H), 1.18-1.14 (m, 6H). ^{13}C NMR ($CDCl_3$, 100MHz, ppm, δ) 192.39, 161.60, 157.15, 150.57, 143.68, 126.93, 117.27, 109.83, 104.06, 97.98, 45.04, 12.34. ESI-MS: *m/z* calculated for $C_{14}H_{16}NO_3$ $[M+H]^+$: 246.1; observed: 246.1

(ii) Synthesis of Compound 2 (NO_2-CM-OH). Compound 1 (245 mg, 1.0 mmol) was dissolved in tetrahydrofuran (THF, 2 mL). In an ice-water bath, a solution of nitromethane (610 mg, 10 mmol) in THF (2 mL) was added dropwise. Then, a solution of *N,N,N'N'*-tetramethylethylenediamine (35 mg, 0.3 mmol) in THF (2 mL) was added dropwise. The reaction mixture was allowed to warm up to ambient temperature and stirred for another 4

h. The solvent was removed by a rotatory evaporator. The residue was re-dissolved in a small amount of dichloromethane and purified by silica gel chromatography (dichloromethane: ethyl acetate 10:1) to give the product as an orange solid (220 mg, 76%). ^{1}H NMR ($CDCl_3$, 400MHz, ppm, δ) 7.42-7.40 (d, 1H), 6.64-6.62 (dd, 1H), 6.54 (s, 1H), 6.38 (s, 1H), 5.82-5.80 (m, 1H), 4.66-4.59 (m, 2H), 3.47-3.41 (m, 4H), 3.18-3.17 (d, 1H), 1.24-1.21 (t, 6H). ^{13}C NMR ($CDCl_3$, 100MHz, ppm, δ) 161.97, 156.50, 151.84, 150.84, 123.95, 109.12, 106.64, 104.96, 98.13, 79.70, 67.06, 44.82, 12.41. ESI-MS: *m/z* calculated for $C_{15}H_{19}N_2O_5$ $[M+H]^+$: 307.1; observed: 307.1

(iii) Synthesis of Compound 3 (NH_2-CM-OH). Compound 2 (920 mg, 3.2 mmol) was suspended in acetic acid (15 mL). The suspension was placed in an ice-water bath. Then zinc powder (1.35 g, 20.8 mmol) was added. After 30 min, the reaction mixture was allowed to warm up to ambient temperature and stirred for another 2 h. The solid was removed by filtration. The filtrate was neutralized by saturated Na_2CO_3 aqueous solution. The mixture was extracted with dichloromethane (200 mL) and then washed with saturated Na_2CO_3 aqueous solution (100 mL) and saturated NaCl aqueous solution (100 mL × 2). The organic phase was dried by anhydrous Na_2SO_4 and concentrated by vacuum. The residue was purified by silica gel chromatography (dichloromethane: methanol 5:1) to give the product as a yellow solid (550 mg, 65%). ^{1}H NMR ($CDCl_3$, 400MHz, ppm, δ) 7.38-7.36 (d, 1H), 6.53-6.52 (dd, 1H), 6.43 (s, 1H), 6.30 (s, 1H), 5.03-5.01 (m, 1H), 3.44-3.34 (m, 6H), 3.16-3.13 (m, 1H), 2.87-2.85 (m, 1H), 1.17-1.15 (t, 6H). ^{13}C NMR ($CDCl_3$, 100MHz, ppm, δ) 162.65, 156.46, 156.30, 150.36, 124.75, 108.66, 106.12, 105.54, 97.75, 68.86, 46.77, 44.47, 44.65, 12.43. ESI-MS: *m/z* calculated for $C_{15}H_{21}N_2O_3$ $[M+H]^+$: 277.2; observed: 277.1.

(iv) Synthesis of Compound 4 (N_3-CM-OH). To a suspension of 4-azidobenzoic acid (65 mg, 0.40 mmol) in dichloromethane (2 mL) in an ice-water bath, triethylamine (80 mg, 0.8 mmol) was added, followed by HATU (170 mg, 0.44 mmol). After 5 min, Compound 3 (100 mg, 0.36 mmol) in dichloromethane (5 mL) was added. The mixture was then stirred for 2 h at ambient temperature. Dichloromethane (80 mL) was used to dilute the reaction mixture, which was then washed by saturated NaCl aqueous solution (100 mL × 3). The organic phase was dried by anhydrous Na_2SO_4 and concentrated using a rotatory evaporator. The residue was purified by silica gel chromatography (dichloromethane: methanol 20:1) to give the product as a yellow solid (130 mg, 86%). ^{1}H NMR ($CDCl_3$, 500MHz, ppm, δ) 7.83-7.82 (d, 2H), 7.75-7.73 (d, 1H), 7.07-7.05 (d, 2H), 7.05-7.03 (m, 1H), 6.67-6.65 (m, 1H), 6.47-6.46 (d, 1H), 6.37 (s, 1H), 5.32-5.30 (m, 1H), 4.20-4.15 (m, 1H), 3.88-3.86 (d, 1H), 3.44-3.39 (m, 4H), 3.31-3.26 (m, 1H), 1.23-1.20 (t, 6H). ^{13}C NMR ($CDCl_3$, 125MHz, ppm, δ) 167.94, 162.83, 156.31, 156.10, 150.88, 143.82, 129.94, 128.90, 125.05, 119.09, 109.06, 106.02, 105.33, 97.69, 69.15, 46.74, 44.75, 12.46. ESI-MS: *m/z* calculated for $C_{22}H_{24}N_5O_4$ $[M+H]^+$: 422.2; observed: 422.2.

(v) Synthesis of Compound 5 (N_3-CM-tetracaine). To a solution of triphosgene (73 mg, 0.25 mmol) in anhydrous dichloromethane (2 mL) in an ice-water bath, a solution of tetracaine (190 mg, 0.72 mmol) and *N,N*-diisopropylethylamine (100 mg, 0.77 mmol) in dichloromethane (5 mL) was added dropwise. The reaction mixture was stirred for 1 h at ambient temperature. The resulted solution was withdrawn in a syringe and then added into a solution of Compound 4 (120 mg, 0.28 mmol) and 1,8-diazabicyclo[5.4.0]undec-7-ene

(250 mg, 1.67 mmol) in dichloromethane (5 mL). The reaction mixture then was stirred for 7 days. Dichloromethane (100 mL) was used to dilute the reaction mixture. The solution was washed by saturated NaCl aqueous solution (100 mL × 3). The organic phase was dried by anhydrous Na_2SO_4 and concentrated by vacuum. The residue was re-dissolved in a small amount of dichloromethane and purified by silica gel chromatography (dichloromethane: methanol 10:1) to give the product as a light-yellow solid (38 mg, 19%). ^{1}H NMR ($CDCl_3$, 500MHz, ppm, *δ*) 8.07-8.05 (d, 2H), 7.77-7.75 (d, 2H), 7.73-7.72 (d, 1H), 7.30-7.28 (d, 2H), 7.08-7.06 (d, 2H), 6.63-6.61 (dd, 1H), 6.45 (s, 1H), 6.25-6.23 (m, 1H), 6.80-5.70 (m, 1H), 4.54-4.52 (t, 2H), 3.94-3.64 (m, 4H), 3.41-3.37 (m, 4H), 3.08-3.05 (m, 2H), 2.60 (s, 6H), 1.48-1.26 (m, 4H), 1.20-1.17 (t, 3H), 0.84-0.82 (t, 3H). ^{13}C NMR ($CDCl_3$, 125MHz, ppm, δ) 166.97, 165.53, 162.46, 156.37, 153.90, 152.99, 150.90, 145.78, 143.60, 130.91, 130.15, 128.92, 127.14, 125.38, 119.05, 109.37, 105.69, 104.62, 97.55, 71.53, 65.84, 60.48, 57.29, 50.19, 44.75, 30.22, 19.78, 15.27, 13.65, 13.41. ESI-MS: *m/z* calculated for $C_{38}H_{46}N_7O_7$ $[M+H]^+$: 712.3; observed: 712.3.

(vi) Synthesis of Compound 6 (P407-yne). P407 (1.2 g) was dissolved in anhydrous THF (10 mL), then NaH (24 mg, 1 mmol) was added at 0 ºC. The mixture was stirred for 2 h. Propargyl bromide (100 uL 80wt% in toluene) was added dropwise. The reaction was stirred overnight. A few drops of water were added dropwise to quench the reaction. Then, the mixture was pass through a short silica gel column (dichloromethane: methanol 2:1). The solution was concentrated and precipitated into cold diethyl ether. The product was collected by filtration as an off-white solid in 90% yield. ^{1}H NMR ($CDCl_3$, 500MHz, ppm, *δ*) 4.21 (m, 4H), 3.77-3.36 (m, 1050H), 2.45 (m, 2H), 1.16-1.12 (m, 180H). GPC: M_n = 12.6 kg/mol, $Đ$ = 1.40.

(vii) Synthesis of Compound 7 (P407-CM-T). Copper (II) sulfate pentahydrate (10 mg, 0.06 mmol) was dissolved in water (0.5 mL), then (+)-sodium L-ascorbate (20 mg, 0.1 mmol) was added. After 5 min, the mixture was added to a methanol solution (10 mL) containing Compound 6 (P407-yne, 200 mg, 0.013 mmol) and Compound 5 (N_3-CM-tetracaine, 40 mg, 0.056 mmol). After 12 h, the mixture was diluted by dichloromethane (100 mL) and washed with saturated NaCl aqueous solution (100 mL × 3). The organic phase was dried by anhydrous Na_2SO_4 and concentrated by vacuum. The residue was purified by silica gel chromatography (dichloromethane: methanol 8:1). The collected solution was concentrated by a rotatory evaporator and precipitated in cold ether. The product was collected by filtration as a light-yellow solid (180, 85%). ^{1}H NMR ($CDCl_3$, 400MHz, ppm, *δ*) 8.23-8.24 (m, 2H), 8.15-8.13 (m, 4H), 8.04-8.03 (m, 4H), 7.89-7.87 (m, 6H), 7.35-7.34 (m, 4H), 6.70-6.68 (m, 2H), 6.49-6.48 (m, 2H), 6.31-6.29 (m, 2H), 5.92-5.81 (m, 2H), 5.59-5.57 (m, 2H), 4.79-4.68 (m, 4H), 4.25-3.20 (m, 1060H), 1.58-0.82 (m, 190H). GPC: M_n = 13.6 kg/mol, $Đ$ = 1.41.

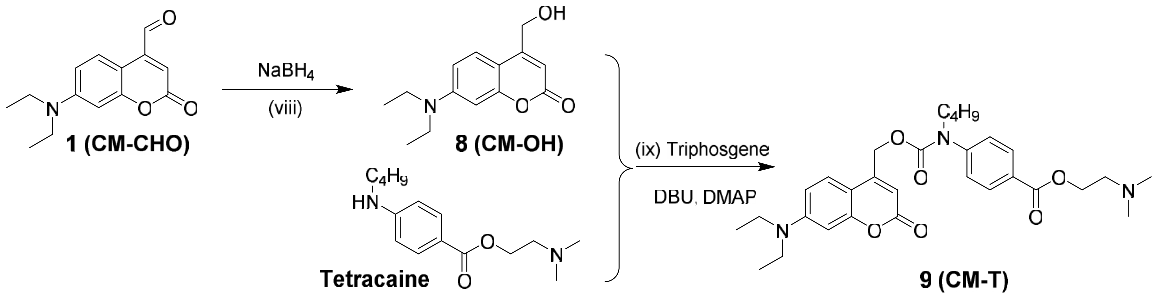

Supplementary Figure 2. Synthetic route to CM-T.

(viii) Synthesis of Compound 8 (CM-OH). Compound 1 (350 mg, 1.43 mmol) was dissolved in methanol (5 mL). Sodium borohydride (170 mg, 4.50 mmol) was added slowly into the above solution in an ice-water bath. After 2 h, water (1 mL) was added to quench the reaction. Then, the mixture was diluted with dichloromethane (100 mL), followed by washed with saturated NaCl aqueous solution (100 mL × 3). The organic phase was dried by anhydrous Na_2SO_4 and concentrated by vacuum. The residue was purified by silica gel chromatography (dichloromethane: ethyl acetate 5:1) to give the product as a yellow solid (250 mg, 71%). ^{1}H NMR ($CDCl_3$, 500MHz, ppm, δ) 7.33-7.31 (d, 1H), 6.58-6.57 (dd, 1H), 6.51-6.50 (d, 1H), 6.27 (s, 1H), 4.84 (s, 2H), 3.43-3.39 (q, 4H), 1.23-1.20 (t, 6H). ^{13}C NMR ($CDCl_3$, 125MHz, ppm, δ) 162.52, 156.17, 154.59, 150.49, 124.36, 108.53, 106.29, 105.48, 97.77, 60.99, 44.72, 12.45. ESI-MS: *m/z* calculated for $C_{14}H_{18}NO_3$ $[M+H]^+$: 248.1; observed: 248.1

(ix) Synthesis of Compound 9 (CM-T). To a solution of triphosgene (36 mg, 0.12 mmol) in anhydrous dichloromethane (2 mL) in an ice-water bath, a solution of tetracaine (90 mg, 0.34 mmol) and triethylamine (50 mg, 0.50 mmol) in dichloromethane (5 mL) was added dropwise. The reaction mixture was stirred for 1 h at ambient temperature. The resulted solution was withdrawn in a syringe and then added into a solution of Compound 8 (65 mg, 0.26 mmol) and triethylamine (120 mg, 1.2 mmol) in dichloromethane (5 mL). After 24 h, the reaction mixture was diluted by dichloromethane (100 mL) and washed by saturated NaCl aqueous solution (100 mL × 3). The organic phase was dried by anhydrous Na_2SO_4 and concentrated by vacuum. The residue was purified by silica gel chromatography (dichloromethane: methanol 20:1) to give the product as a yellow solid (60 mg, 45%). ^{1}H NMR ($CDCl_3$, 500MHz, ppm, δ) 8.07-8.05 (d, 2H), 7.32-7.30 (d, 2H), 7.24-7.22 (d, 1H), 8.54-8.51 (dd, 1H), 6.49-6.48 (d, 1H), 5.91 (m, 1H), 5.25 (s, 2H), 4.44-4.41 (t, 2H), 3.76-3.72 (t, 2H), 3.42-3.37 (q, 4H), 2.73-2.70 (t, 2H), 2.33 (s, 6H), 1.56-1.50 (m, 2H), 1.34-1.28 (m, 2H), 1.21-1.18 (t, 6H), 0.90-0.86 (t, 3H) ^{13}C NMR ($CDCl_3$, 125MHz, ppm, δ) 165.80, 161.83, 159.19, 154.18, 150.61, 149.91, 131.63, 130.68, 128.70, 124.36, 108.56, 106.08, 105.91, 97.77, 63.05, 62.79, 57.76, 50.29, 45.78, 44.73, 30.36, 29.69, 19.84, 13.71, 12.42. ESI-MS: *m/z* calculated for $C_{30}H_{40}N_3O_6$ $[M+H]^+$: 538.3; observed: 538.3.

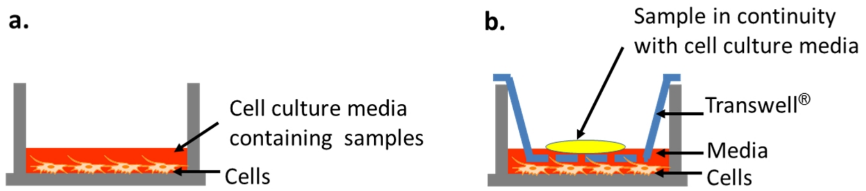

Supplementary Figure 3. Illustration of two kind of cytotoxicity experiments: **a**. samples were dissolved in the cell culture media, and **b**. samples were placed in Transwell® inserts such that they were in continuity with the cell culture media.

Supplementary Discussion

Oxidation of 7-Diethylamino-4-methylcoumarin by SeO_2 produced CM-CHO,[2] which had a characteristic peak of aldehyde at about 10 ppm in ^1H NMR (Supplementary Figure 4). After reacted with nitromethane, the aldehyde peak disappeared, and protons near the nitro group in NO_2-CM-OH appeared between 4 to 5 ppm (Supplementary Figure 5),[1] which shifted to lower chemical shift after the nitro group was reduced into amine group by zinc (Supplementary Figure 6). Then, 4-azidobenzoic acid was used to selectively react with the amino group in NH_2-CM-OH under the reagent HATU. The peaks corresponding to the benzene ring in 4-azidobenzoic acid ester was clearly displayed in N_3-CM-OH (Supplementary Figure 7). Then, tetracaine was reacted with triphosgene to produce tetracaine chloroformate, which could react with the hydroxyl group in N_3-CM-OH to give N_3-CM-tetracaine. The proton near the hydroxyl group at 5.3 ppm shift to high chemical shift after the formation of the carbamate bond (Supplementary Figure 8). To prepare P407-yne, the hydroxyl end groups of P407 was deprotonated by strong base sodium hydride, followed by substituted with propargyl bromide. The appearance of the alkynyl peak at about 2.5 ppm (Supplementary Figure 9) indicated the successful modification of P407. Then, P407-yne and N_3-CM-tetracaine underwent copper-catalyzed azide-alkyne cycloaddition (CuAAC). The copper salt was removed by passing a silica gel column, and organic small molecules were removed by precipitation. The signals from P407, coumarin and tetracaine moieties were clearly identified in the spectrum of P407-CM-T (Supplementary Figure 10).

In order to compare with the polymer counterpart, small molecular conjugates of coumarin and tetracaine was also prepared. CM-CHO was directly reduced to CM-OH (Supplementary Figure 2). In the NMR spectra, the aldehyde signal at 10 ppm disappeared, and the new signal at 4.8 ppm (Supplementary Figure 11) can be attributed to the proton near the newly formed hydroxyl group. Then, CM-OH was reacted with the freshly prepared tetracaine chloroformate to give CM-T. The NMR signal at 4.8 ppm shifted to 5.2 ppm (Supplementary Figure 12) indicated the formation of the carbamate bond.

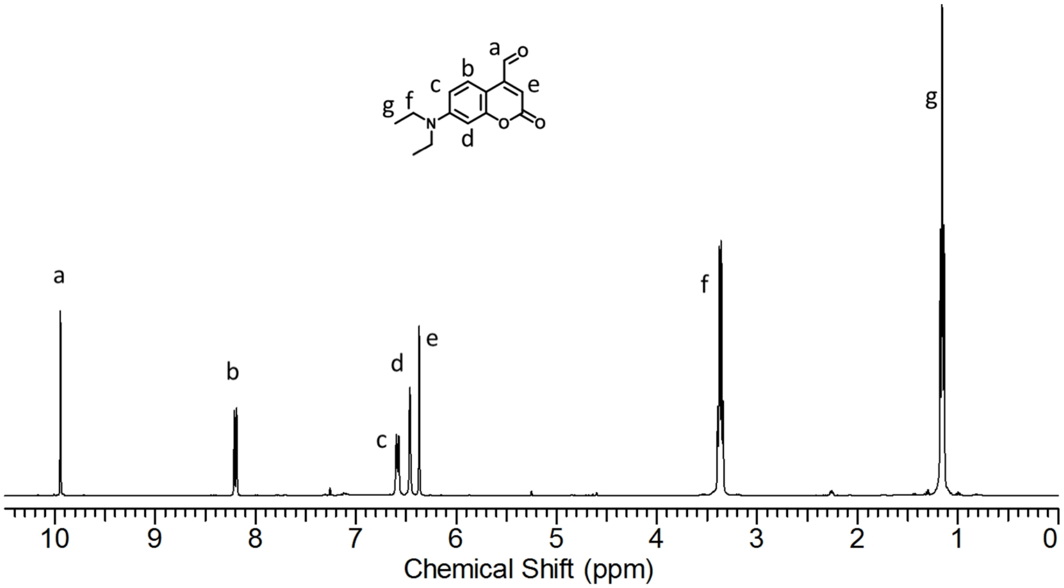

Supplementary Figure 4. ^{1}H NMR spectrum of Compound 1 (CM-CHO).

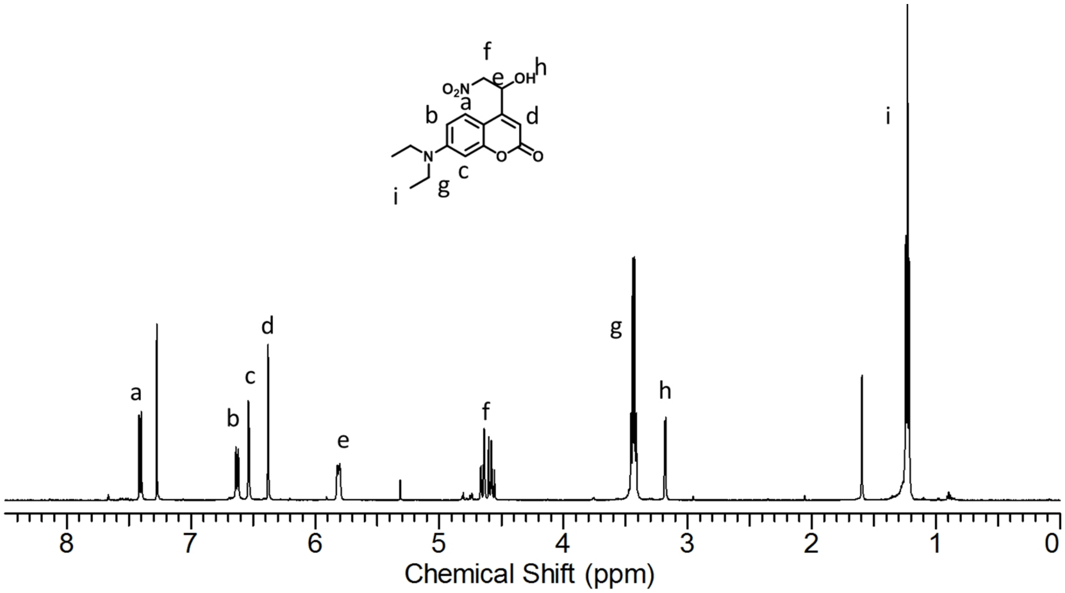

Supplementary Figure 5. ^{1}H NMR spectrum of Compound 2 (NO_2-CM-OH).

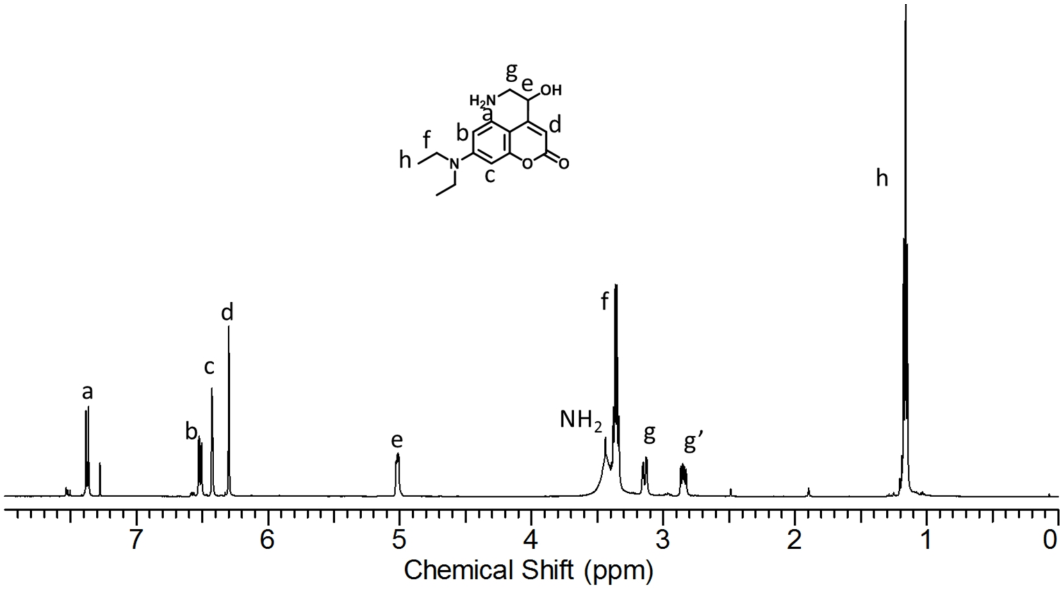

Supplementary Figure 6. ^{1}H NMR spectrum of Compound 3 (NH_2-CM-OH).

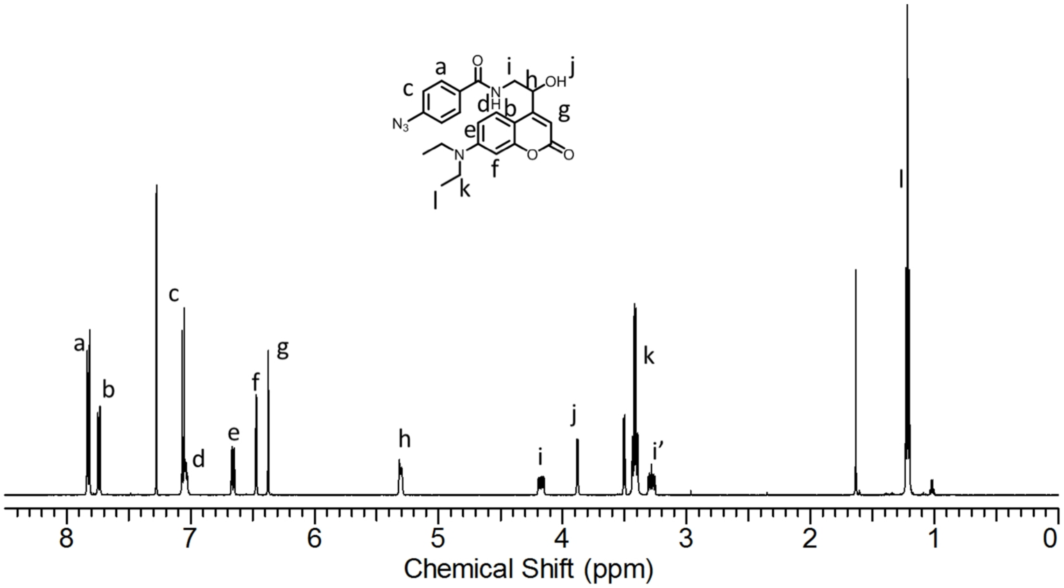

Supplementary Figure 7. ^{1}H NMR spectrum of Compound 4 (N_3-CM-OH).

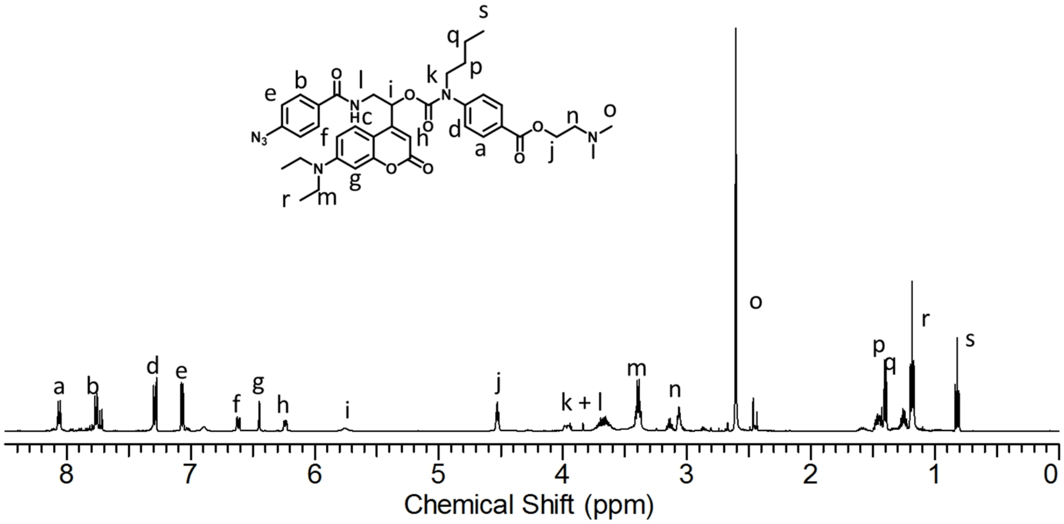

Supplementary Figure 8. ^{1}H NMR spectrum of Compound 5 (N_3-CM-tetracaine).

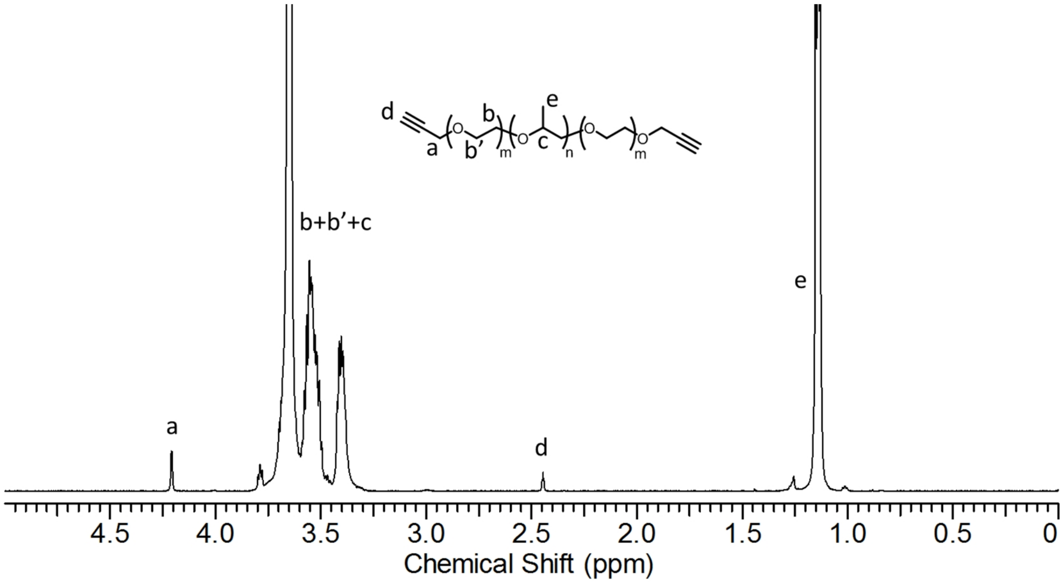

Supplementary Figure 9. ^{1}H NMR spectrum of Compound 6 (P407-yne).

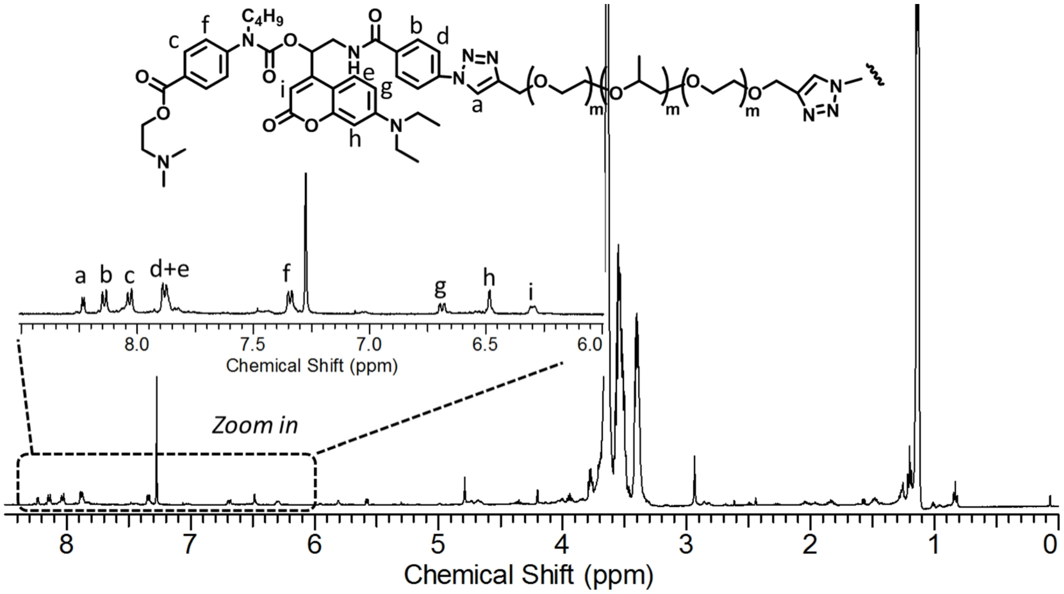

Supplementary Figure 10. ^{1}H NMR spectrum of Compound 7 (P407-CM-T).

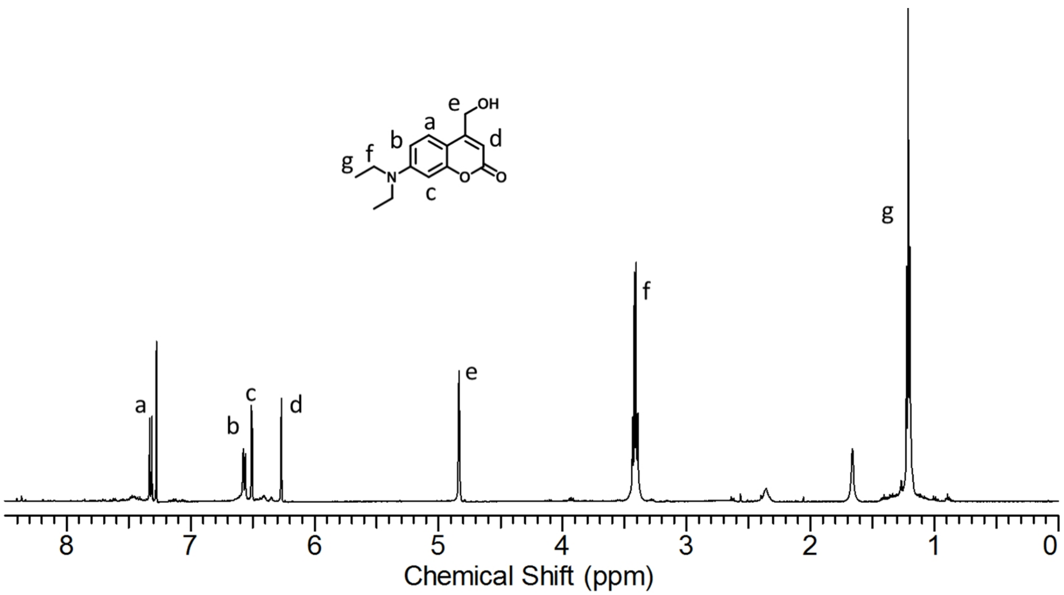

Supplementary Figure 11. ^{1}H NMR spectrum of Compound 8 (CM-OH).

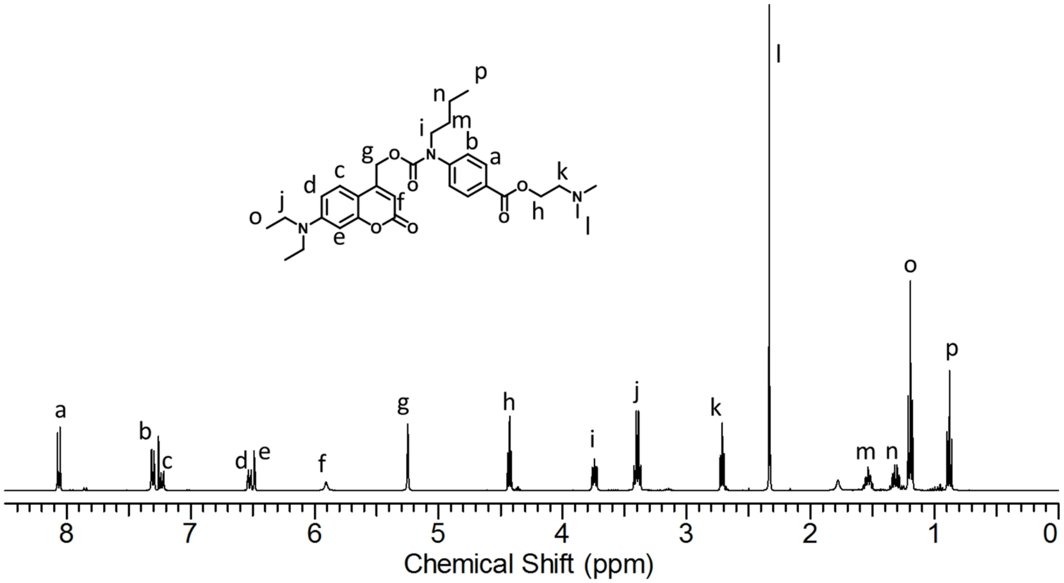

Supplementary Figure 12. ^{1}H NMR spectrum of Compound 9 (CM-T).

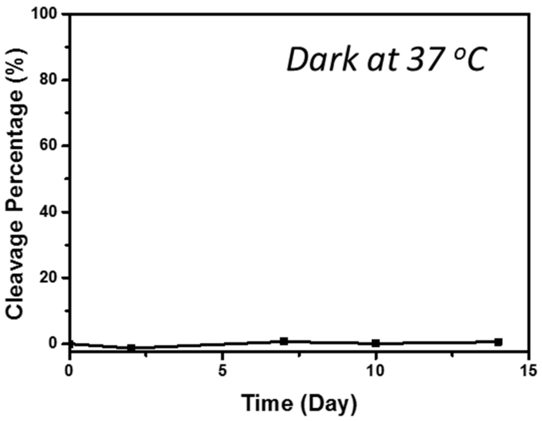

Supplementary Figure 13. Stability of P407-CM-T in the dark. Data are means ± SD (n = 4)

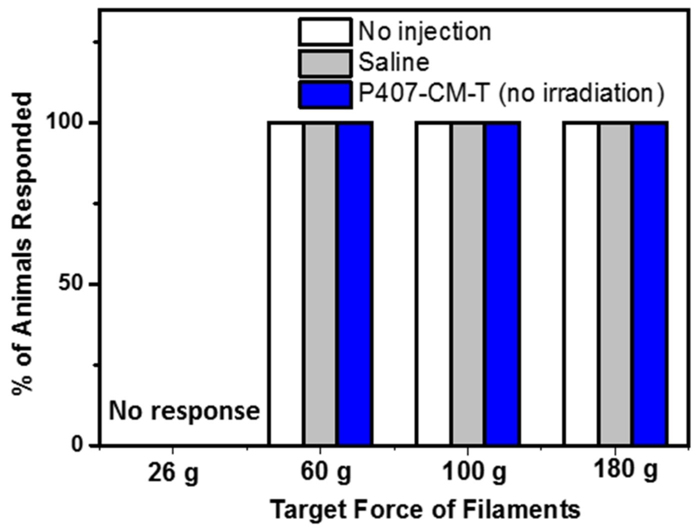

Supplementary Figure 14. Percentage of animals (n = 5) responding to filaments.

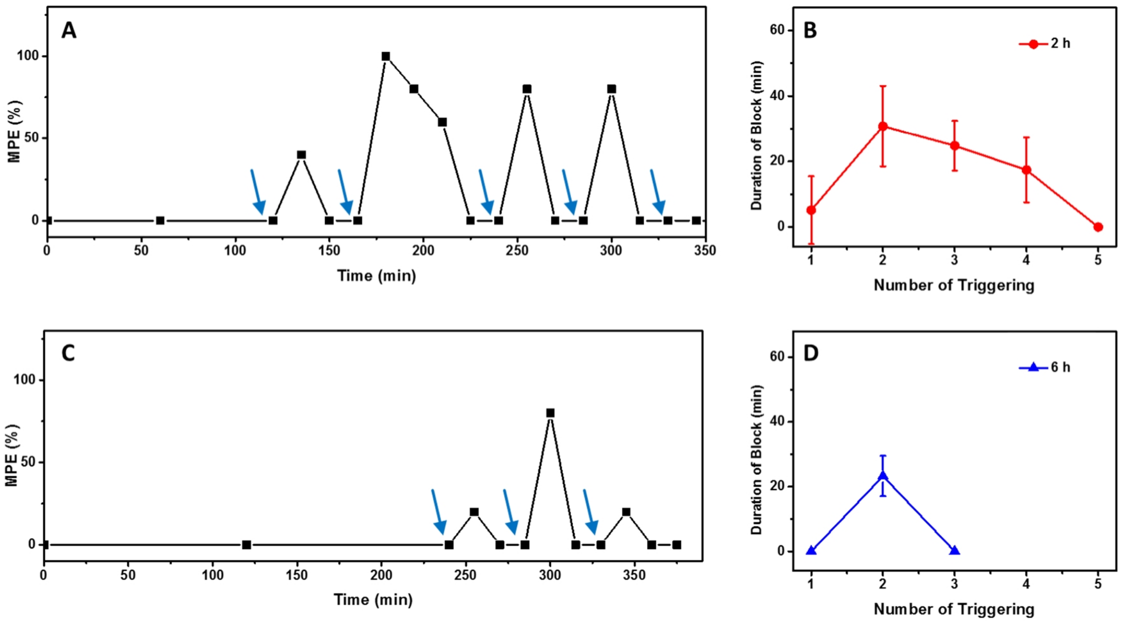

Supplementary Figure 15. A. Representative time course of nerve block with multiple light triggering events with the first one performed 2 hours after injection and B. the average duration of block after each triggering event. C. Representative time course of nerve block with multiple light triggering events with the first one performed 6 hours after injection and D. the average duration of block after each triggering event. (blue arrows represent LED triggering for 2 min at 300 mW cm^{-2}). Data are means ± SD (n=4). Source data are provided as a Source Data file.

Supplementary Table 1. Duration of block of P407-CM-T after repeatedly irradiation at 300 mW cm^{-2} for 2 min

Time of first irradiation	Irradiation Event #	Duration of Block (min)
0 h after injection	1	36.4 ± 8
	2	46.1 ±13.8
	3	29.8 ±8.3
	4	20.6 ±8.9
	5	0
2 h after injection	1	5.2 ± 10.3
	2	30.7 ± 12.3
	3	24.8 ± 7.6
	4	17.4 ± 9.9
	5	0
6 h after injection	1	0
	2	23.3 ± 6.2
	3	0

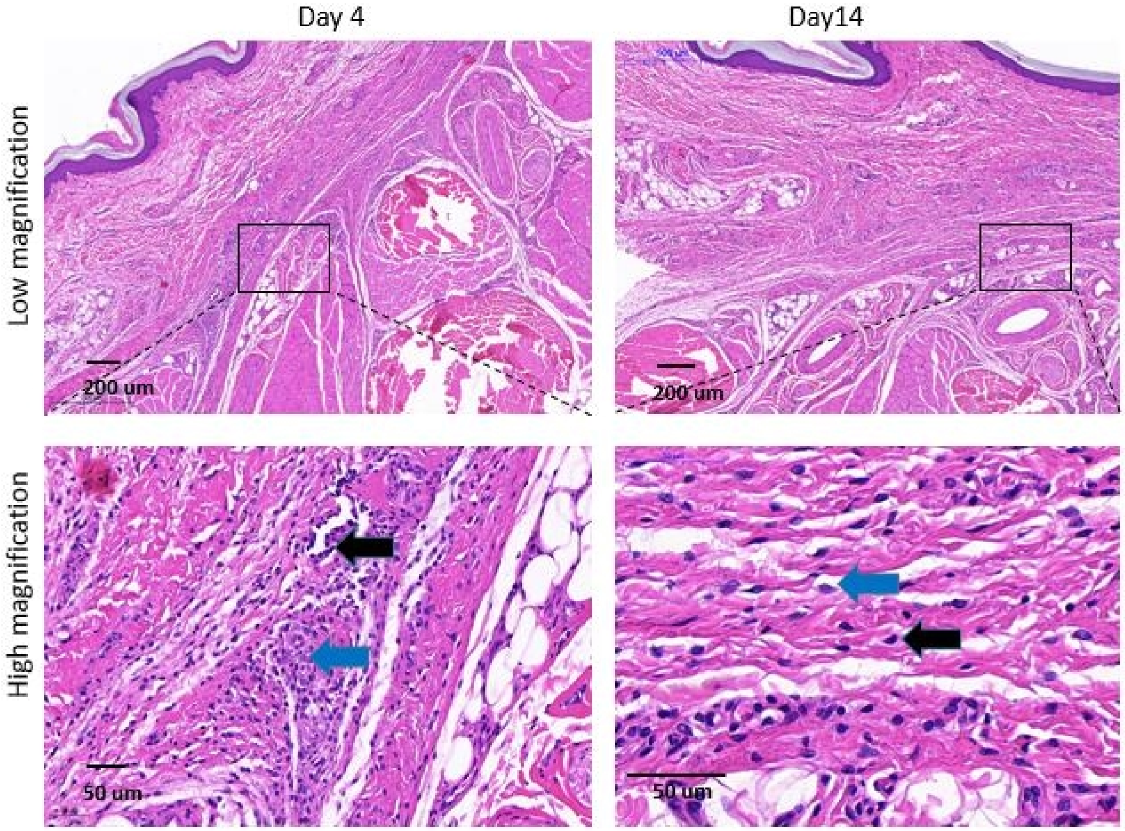

Supplementary Figure 16. Tissue reaction to rat foot pad injections of P407 on days 4 and 14 after injection. Black arrows: lymphocytes. Blue arrows: macrophages. Panels on bottom (scale bar: 50 µm) are magnified views of the outlined sections in the panels on the top (scale bar: 200 µm). Data are representative of 4 animals.

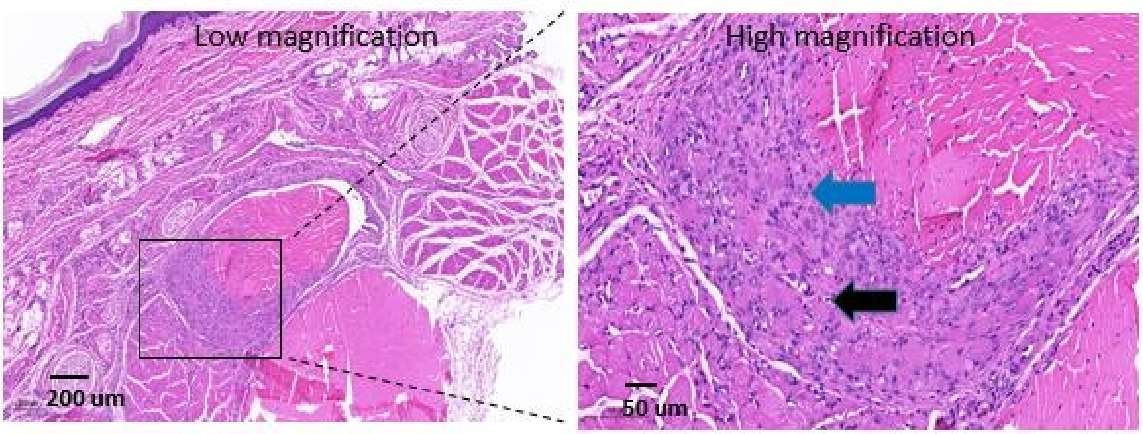

Supplementary Figure 17. Tissue reaction to rat foot pad injections of tetracaine on day 14 after injection. Black arrows: lymphocytes. Blue arrows: macrophages. Panel on right (scale bar: 50 µm) is a magnified view of the outlined section in the panel on the left (scale bar: 200 µm). Data are representative of 4 animals.

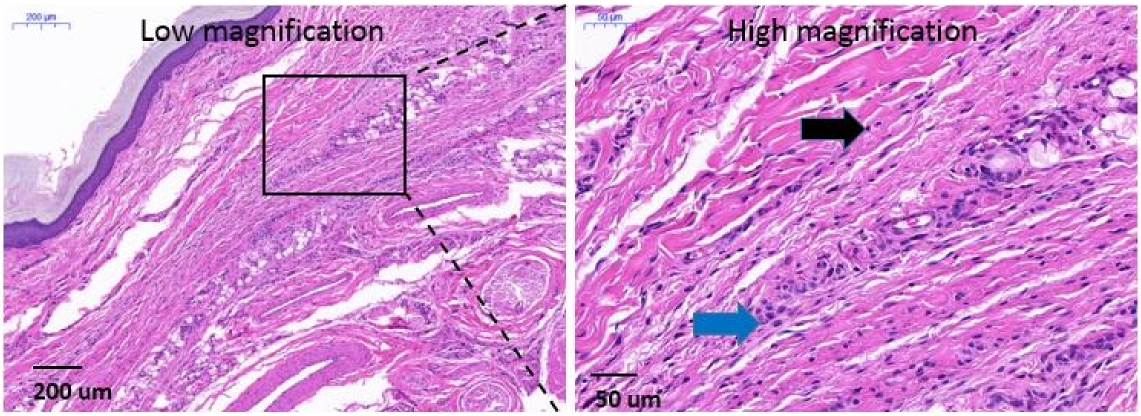

Supplementary Figure 18. Tissue reaction to rat foot pad injections of P407-CM-T (without irradiation) on day 14 after injection. Black arrows: lymphocytes. Blue arrows: macrophages. Panel on right (scale bar: 50 µm) is a magnified view of the outlined section in the panel on the left (scale bar: 200 µm). Data are representative of 4 animals.

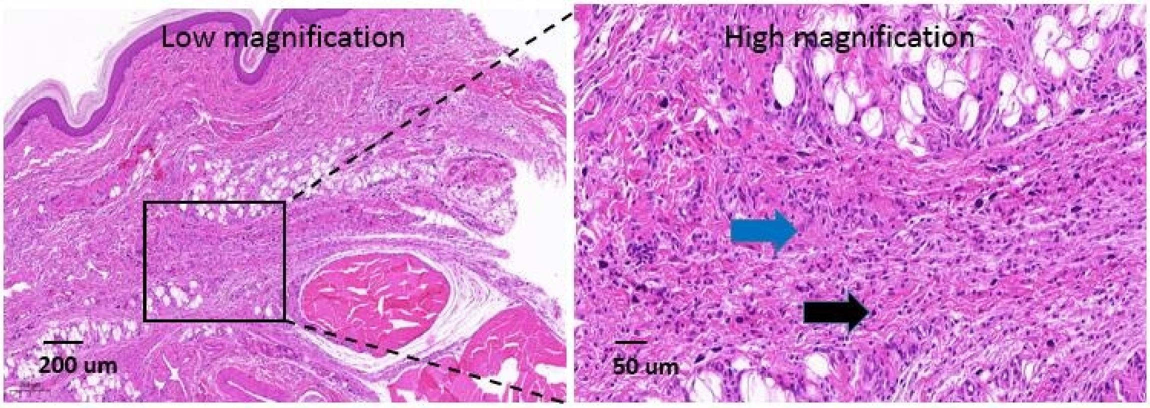

Supplementary Figure 19. Tissue reaction to rat foot pad injections of P407-CM-T (with irradiation on the first day) on day 14 after injection. Black arrows: lymphocytes. Blue arrows: macrophages. Panel on right (scale bar: 50 µm) is a magnified view of the outlined section in the panel on the left (scale bar: 200 µm). Data are representative of 4 animals.

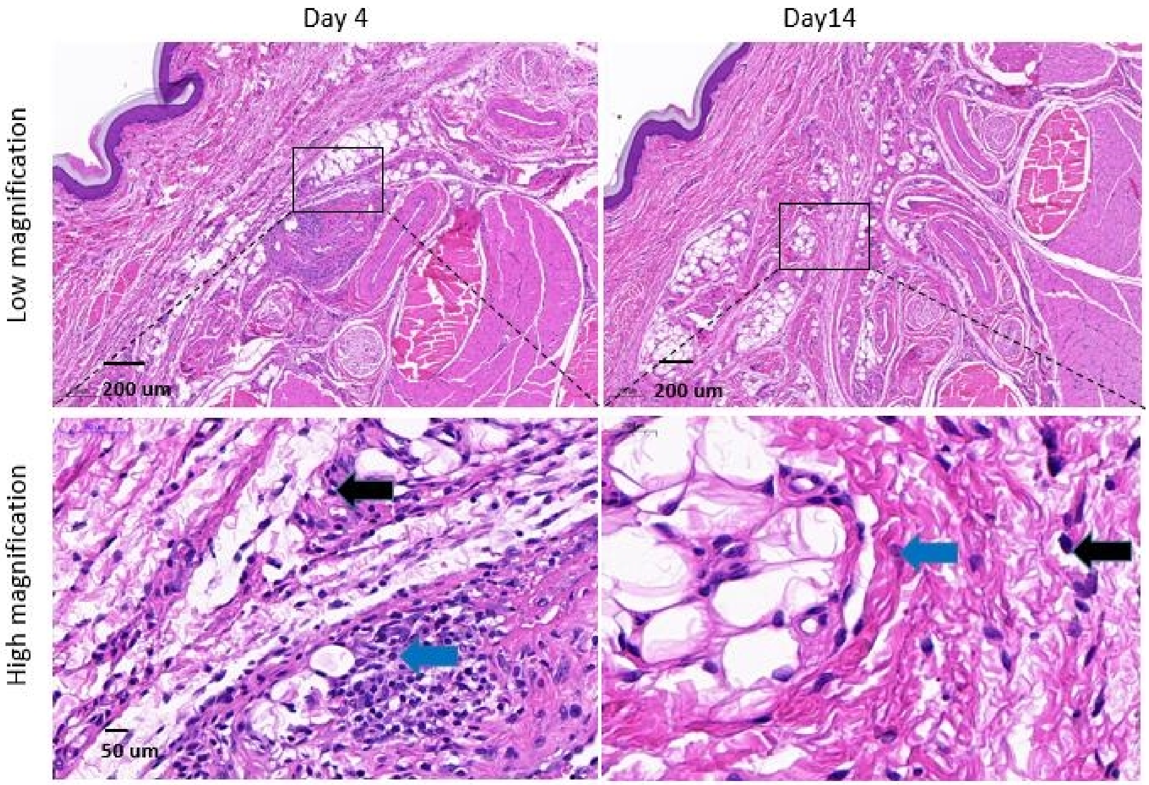

Supplementary Figure 20. Tissue reaction to rat foot pad injections of saline on days 4 and 14 after injection. Black arrows: lymphocytes. Blue arrows: macrophages. Panels on bottom (scale bar: 50 µm) are magnified views of the outlined sections in the panels on the top (scale bar: 200 µm). Data are representative of 2 animals.

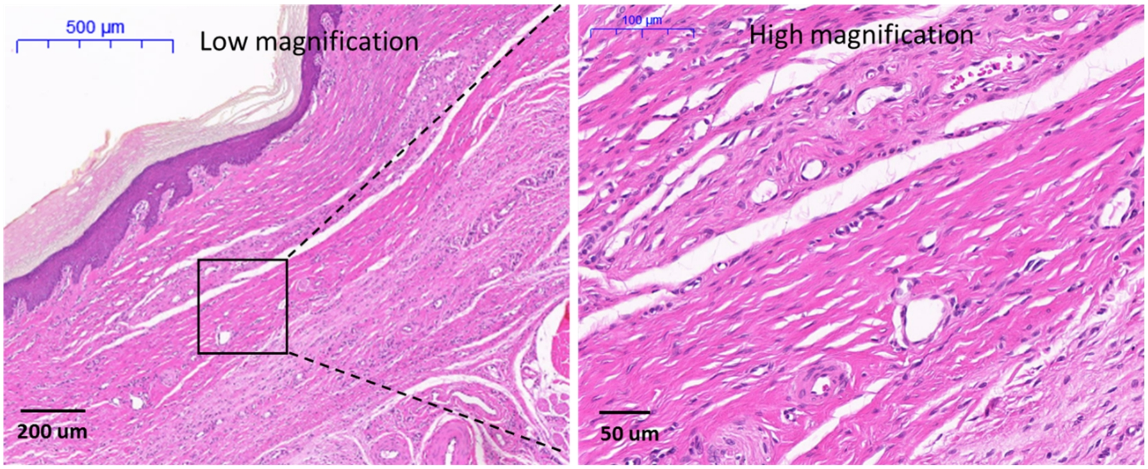

Supplementary Figure 21. Tissue reaction 4 days after irradiation (300 mW/cm^2, 2 min) without injection of test materials. Panel on right (scale bar: 50 μm) is a magnified view of the outlined section in the panel on the left (scale bar: 200 μm). Data are representative of 2 animals.

Supplementary References

1. Yamazoe, S., Liu, Q., McQuade, L.E., Deiters, A. & Chen, J.K. Sequential Gene Silencing Using Wavelength-Selective Caged Morpholino Oligonucleotides. *Angew. Chem. Int. Ed. Engl.* **53**, 10114-10118 (2014).
2. Lin, W., Long, L., Feng, J., Wang, B. & Guo, C. Synthesis of meso-Coumarin-Conjugated Porphyrins and Investigation of Their Luminescence Properties. *Eur. J. Org. Chem.* **2007**, 4301-4304 (2007).
